# Comparison Between the Impact of Diabetes Mellitus on Liver Diseases and Vice Versa Among Saudi and Egyptian Patients

**DOI:** 10.3390/healthcare13040376

**Published:** 2025-02-10

**Authors:** Madiha R. Mahmoud, Somia Ibrahim, Mona M. Shahien, Amal Daher Alshammari, Fahaad S. Alenazi, Fayez Alreshidi, Ahmed Aljadani, Ashraf Abdel Khalik, Abeer H. Elhaj, Amany M. Khalifa, Hend Faleh Alreshidi, Hemat El-Sayed El-Horany, Kamaleldin B. Said, Marwa H. Abdallah, Amna A. Metwaly

**Affiliations:** 1 Department of Pharmacology, College of Medicine, University of Ha’il, Ha’il 81422, Saudi Arabia; fs.alenazi@uoh.edu.sa; 2 Department of Pediatrics, College of Medicine, University of Ha’il, Ha’il 81422, Saudi Arabia; somaiabashir@yahoo.com (S.I.); m.shahin@uoh.edu.sa (M.M.S.); 3 Department of Family and Community Medicine, College of Medicine, University of Ha’il, Ha’il 81422, Saudi Arabia; amal.alshammari@uoh.edu.sa (A.D.A.); fs.alreshidi@uoh.edu.sa (F.A.); beero.work@gmail.com (A.H.E.); hendfal@hotmail.com (H.F.A.); 4 Department of Psychiatry, College of Medicine, University of Ha’il, Ha’il 81422, Saudi Arabia; a.aljadani@uoh.edu.sa; 5 Department of Intensive Care Unit, TBRI, Ministry of Higher Education and Scientific Research, Giza 12411, Egypt; dr.ashraf.a@hotmail.com (A.A.K.); amnametwaly@hotmail.com (A.A.M.); 6 Medical Parasitology, Pathology Department, College of Medicine, University of Ha’il, Ha’il 81422, Saudi Arabia; a.khalifa@uoh.edu.sa; 7 Medical Parasitology Department, Faculty of Medicine, Alexandria University, Alexandria 21526, Egypt; 8 Department of Biochemistry, College of Medicine, University of Ha’il, Ha’il 81422, Saudi Arabia; h.elhorany@uoh.edu.sa; 9 Medical Biochemistry Department, Faculty of Medicine, Tanta University, Tanta 31527, Egypt; 10 Department of Pathology and Microbiology, College of Medicine, University of Ha’il, Ha’il 81422, Saudi Arabia; kbs.mohamed@uoh.edu.sa; 11 Genomics, Bioinformatics and Systems Biology, Carleton University, 1125 Colonel-By Drive, Ottawa, ON K1S 5B6, Canada; 12 Department of Pharmaceutics, College of Pharmacy, University of Ha’il, Ha’il 81422, Saudi Arabia

**Keywords:** type 2 diabetes, lipid profile, liver diseases, liver enzymes, glycated hemoglobin, Saudi patients, Egyptian patients

## Abstract

**Background**: The risk of dying from chronic liver diseases (CLDs) is two to three times higher for patients with diabetes (DM). Nonalcoholic fatty liver disease (NAFLD) is the primary cause of this increased risk, which has an etiology unrelated to alcohol or viruses. Previous research reported that diabetes and CLD are related, since they influence each other. **Aim**: Estimation of the impact of diabetes (DM) on liver diseases (LD), and of the impact of liver diseases on DM among Egyptian and Saudi patients. It is a descriptive and prospective analytical study design. The investigation was carried out in Saudi Arabia and Egypt at gastroenterology outpatient clinics. **Methods**: Prospective data were collected through face-to-face patient interviews during clinic visits between June 2021 and June 2023. The interviews covered the patients’ basic characteristics and information on DM and LD. Certain laboratory tests were conducted on these patients, such as liver function, glucose level, lipid profile, INR, and prothrombin time. **Results**: The total of 2748 participants in this study included 1242 diabetic patients of both genders from Saudi Arabia and 1506 from Egypt. Most Saudis had between 10 and 20 years’ duration of DM (35.5%), with HbA1c (7–10%) values of 47.8%, while the Egyptian patients had >20 years’ duration of DM (39.8%), with HbA1c (7–10%) values of 49.8%. Regarding the impact of DM on the development of liver diseases, about 35.5% (Saudis) vs. 23.5% (Egyptians) had liver diseases due to DM, a significant difference (*p*-value = 0.011). Liver enzymes were increased in many of the Egyptian and Saudi patients (41.4% vs. 33%), while the presence of fatty liver (28.2% vs. 35.7%) and hepatocellular carcinoma (13.7% vs. 6.1%) were also significantly different (*p*-value = 0.047). While the impact of liver diseases on DM was observed more among Egyptian (59%) than among Saudi (46.4%) patients because of liver cirrhosis (HCV or HBV), known to be a reason for diabetes in Egyptians (27.9%) vs. Saudis (8.0%), a higher incidence of fatty liver leading to DM was observed in Saudis than in Egyptians (15.9% vs. 11.6%) (*p*-value = 0.000. Obesity was more prevalent among Saudi patients (63.8%) than among Egyptian patients (48.6%) (*p*-value = 0.019). Fewer Egyptians (about 65%) suffered from dyslipidemia than Saudis (about 80%). Higher INR and longer prothrombin times were observed in Egyptians (29.9% and 29.1%, respectively) than in Saudis (20.3% and 18.8%, respectively), with a significant difference between the two nations (*p*-value < 0.050). **Conclusions**: We may conclude that diabetes in most patients has a negative impact on the development of liver diseases (particularly fatty liver in Saudi patients). In addition, most liver diseases (liver cirrhosis) have a negative influence on the development of DM (more so in Egyptian patients). There is a link between DM and liver disease. In particular, liver cirrhosis and diabetes were found to influence each other. Therefore, correct medication, adherence to treatment, lifestyle modifications, successful cirrhosis control (in patients with liver diseases), and diabetic control (in diabetic patients) could lead to effective management of both diseases. The negative fallouts in the two cases were prompted by obesity, morbid eating, and poor quality of life.

## 1. Introduction

There were an estimated 463 million cases of diabetes globally in 2019, forecast to rise to 10.2% (578 million) by 2030, and to 700 million by 2045 [1]. As a result, it is expected that, by 2030, there will be 54% more diabetic patients, and the death rate will have increased by 38%. Also, advanced treatment allows patients to survive longer, which could result in higher prevalence [2]. More than 25% of Saudi Arabia’s adult population is estimated to be suffering, and by 2030, that percentage is expected to more than double [3]. Many vascular complications are present at the diagnosed onset of diabetes. Asian populations have a higher risk of becoming diabetic at a younger age than Western people [4]. Long-term metabolic diseases include microvascular and macrovascular issues, which can lead to damage to several systems. Microvascular problems include retinopathy, nephropathy, and neuropathy, which affect the nervous system and kidneys. Peripheral vascular disease, stroke, and cardiovascular disease are examples of macrovascular problems. Peripheral vascular disease may result in gangrene, amputation, and non-healing bruises or injuries [5].

It is necessary to achieve good metabolic control of diabetes, and pharmacological treatment and a better lifestyle are necessary. Achieving near-normal glycated hemoglobin (HbA1c) significantly decreases the risk of macrovascular and microvascular complications [6]. Recently, the number of hypoglycemic agents available for the treatment of T2DM has increased, according to the American Diabetes Association (ADA) and the European Association for the Study of Diabetes (EASD) [7]. Moreover, changes in lifestyle have proven to be beneficial, but many patients were found to be facing long-term complications due to various experiences and perceptions [8]. Drug therapy is aimed at maintaining glycemic control. Metformin remains the first choice of treatment for most patients. Other alternative or second-line treatment options should be individualized depending on the characteristics of each patient [9]. Robust evidence consistently indicates that diabetes self-management education and support (DSMES) enhances knowledge, glycemic control, and both clinical and psychological outcomes, while also decreasing hospital admissions and overall mortality and demonstrating cost-effectiveness [10].

The liver is vital for glucose homeostasis and other body metabolic activities, as it stores glycogen, produces glucose through the processes of gluconeogenesis and glycogenolysis, and controls the blood sugar level through feeding and fasting [11]. Those with liver disease may experience hyperglycemia after eating foods high in carbs [12]. During feeding, the liver absorbs glucose, and then converts excess glucose to glycogen, which is stored in the liver; lipogenesis then converts it into triacylglycerols. These processes are tied to insulin, which plays a critical role in reducing glycogenolysis [13].

An estimated 2 million people die worldwide each year of liver disease: complications from cirrhosis account for 50% of these deaths, and viral hepatitis and hepatocellular cancer for 50%. Liver cancer is the 16th most common cause of death worldwide, while cirrhosis is ranked 11th; these two conditions account for 3.5% of all deaths worldwide [8]. In the developed countries, nonalcoholic fatty liver disease (NAFLD) is now the most common cause of chronic liver disease (CLD) and a key candidate for liver transplantation. The prevalence of NAFLD exceeds an estimated 40% in the Americas and Southeast Asia [14]. In the US, the incidence of NAFLD has also raised the prevalence of risk factors associated with NAFLD, such as obesity, dyslipidemia, insulin resistance, and hypertension. Many studies have shown that keeping a healthy lifestyle, losing weight, and actively controlling each component of the metabolic syndrome may help prevent, delay, or even repair liver damage due to NAFLD [15]. Screening for NAFLD can help in the early identification and prevention of hepatic and extrahepatic impacts in high-risk subpopulations, such as those with T2DM and other metabolic risk factors. NAFLD raises the risk of premature cardiovascular disease and associated death [16]. Liver dysfunction and scarring can happen over time due to NAFLD. According to at least one estimate, between 6 and 8 million Americans suffer from NASH with mild to severe liver scarring. Rezdiffra partially activates a thyroid hormone receptor; when it performs this in the liver, it reduces the accumulation of liver fat [17]. Some studies have indicated a link between DM and CLD, as they influence each other. Therefore, the right medication, lifestyle modifications, and successful cirrhosis control could lead to effective diabetes management [18].

There is growing evidence that people with DM have a much higher chance of death from their condition than people with cirrhosis alone, as well as a higher chance of their fibrosis progressing earlier. Diabetes has been linked to a higher risk of death from cirrhosis and cirrhosis related to hepatitis, whether viral or not [19]. In addition, it has been reported that DM could play an important role in the disease’s progression toward hepatocellular carcinoma (HCC). Because most diabetic drugs are processed in the liver, which alters drug metabolism and increases the risk of hypoglycemia and lactic acidosis, managing diabetes in people with CLD is difficult. Therefore, metformin has demonstrated its efficacy in lowering the risk of HCC, in contrast to sulphonylurea and insulin [20].

Patients with diabetes have a two to three times higher likelihood of dying from CLD. This higher risk is mainly due to NAFLD, an etiology unrelated to viruses or alcohol. The survival of diabetic patients can improve clinically from an early diagnosis and treatment of NAFLD, if present [11]. Hepatic cirrhosis and DM may be related via the same mechanism that causes chronic HCV, hemochromatosis, and hepatic autoimmune disorders [21]. Therefore, this study aimed to estimate both the harmful effects of uncontrolled diabetes that produce liver diseases and the impact of liver diseases that lead to diabetes among Egyptian and Saudi patients.

## 2. Subjects and Methods

### 2.1. Patients

This prospective study was conducted at diabetes, internal medicine, and hematology clinics at Ha’il University Clinics and at the Theodor Bilharz Research Institute (TBRI), as well as at other diabetes clinics in Egypt. All individuals provided informed consent before participation. Data were collected from both males and females visiting the outpatient department over a period of two years (June 2021–June 2023) through face-to-face patient interviews. The remaining data were collected from the electronic health records for inpatients. These include demographic information and all relevant data, such as the area of residence and past medical history about diabetes and liver diseases.

### 2.2. Laboratory Tests

Some laboratory tests for DM and LD were carried out among Saudi and Egyptian patients. Blood samples were drawn under standard infection control guidelines. Fasting serum samples were immediately used for biochemical analyses of glucose, cholesterol, triglycerides, HDL-cholesterol, bilirubin, ALT, AST, and albumin. To separate sera, samples were allowed to clot at room temperature and then centrifuged at 1000× *g* for 20 min. A separate aliquot of K3EDTA blood was also drawn for HbA1c estimation. Glucose levels (fasting and postprandial), cholesterol, triglycerides, HDL-cholesterol, bilirubin, ALT, AST, albumin, and HbA1c were measured using commercial kits (Spinreact, Girona, Spain, Commercial office-Barcelona) using an automated chemistry analyzer (Dimension EXL 200 integrated chemistry system, Siemens, Munich, Germany). LDL-cholesterol was calculated according to Friedewald’s formula [22]. Moreover, citrated blood samples were centrifuged, and plasma was immediately separated and tested for the prothrombin time (PT) and international normalized ratio (INR) using a coagulation analyzer.

Table 1 shows that of 2873 patients with diabetes, 1295 were Saudi and 1578 were Egyptian. Due to their ages (less than 20 years old) and/or incomplete data, 129 (53 Saudi and 76 Egyptian) patients were excluded, as shown in the flow chart of study participants. 

### 2.3. Inclusion Criteria

All patients with chronic liver diseases and/or diabetes mellitus aged 20 and above and of either gender. A total of 2748 diabetic patients participated in this study. Of these, 1242 were from Saudi Arabia and 1506 from Egypt.

### 2.4. Exclusion Criteria

Any patients younger than 20 years old and/or patients with incomplete data.

### 2.5. Statistical Analysis

Statistical analysis was performed using the Statistical Package for Social Sciences (version 25, SPSS Inc., Chicago, IL, USA). Continuous variables were expressed as mean ± SE, and categorical variables were presented using frequencies (n) and percentages (%). A *p*-value ≤ 0.05 was considered statistically significant according to the Pearson chi-square test.

## 3. Results

**Figure 1:**
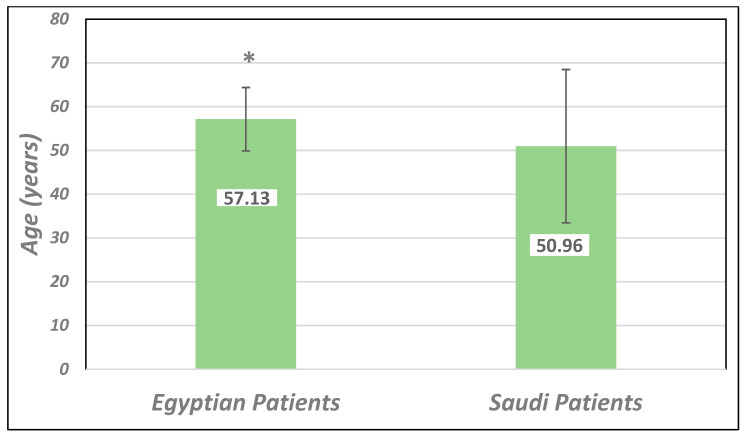
Age (years) of participants in Saudi and Egyptian patients with diabetes (means *±* SD). * Significantly higher ages of Egyptian than of Saudi participants at *p*-value < 0.05.

The study included 2748 (1242 Saudi and 1506 Egyptian) diabetes patients of both genders. There were more female diabetic patients in the KSA. Table 2 and Figure 1 show that among Saudi patients, the average age was 50.96 ± 17.53 (mean ± SD), and the 41–60-year-old group was the most represented (35.5%). The Egyptian participants’ average age was 57.13 ± 7.25, with most being 51 years old or older. The duration of diabetes was significantly different between the two countries at *p*-value = 0.000. In Saudi patients, the duration was 35.5% (>10–20 years), followed by 23.9% (between 5 and 10 years). However, in Egyptian patients, the duration of diabetes was different, about 39.8% (>20 years), followed by 27.1% (between 10 and 20 years).

The lifestyles of patients may be considered risk factors for diabetes, such as smoking, obesity, unhealthy foods, and less exercise. In Table 2, we observed that smoking prevalence was comparable across both nations (about 30% at *p*-value = 0.641). Obesity was more widespread among Saudis (63.8%) than in Egyptians (48.6%), which was significantly different (*p*-value = 0.004). Furthermore, a higher percentage of Saudi patients reported that they did not eat healthy food or engage in regular exercise (69.9% and 63.0%, respectively), compared to lower percentages in their Egyptian counterparts (48.6% and 49.0%, respectively). These results were significantly different between the two nations (*p*-value = 0.000 and 0.008, respectively). Patients’ work duties and activities were affected in 73.2% of Saudi vs. 66.1% of Egyptian patients, not significantly different between the two nations (*p*-value = 0.151).

Table 3 shows that the treatment modalities were significantly different between the two countries (*p*-value = 0.028). Approximately one-third (for each group) of Egyptian patients had insulin injections, hypoglycemic agents alone, or hypoglycemic medications in conjunction with regulatory agents. In contrast, insulin injections were utilized by 33.3% of Saudi patients, followed by hypoglycemic agents combined with regulating agents (31.9%), and then hypoglycemic agents alone (21.7%).

**Figure 2:**
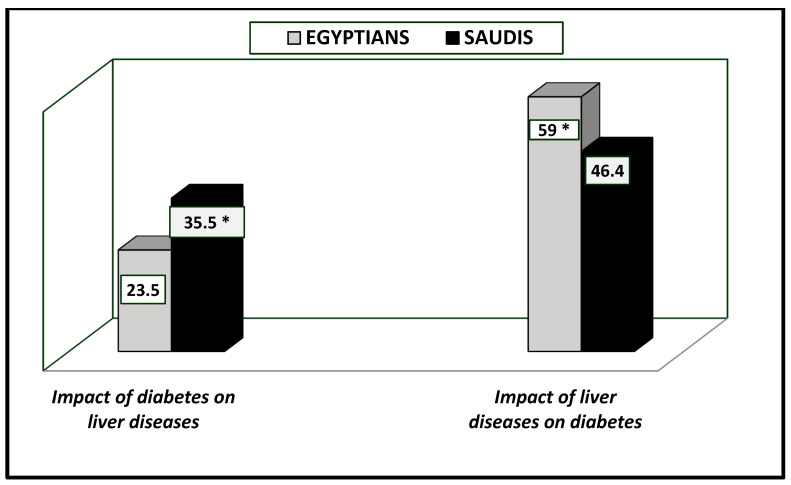
Impact of diabetes on liver diseases and impact of liver diseases on diabetes among Egyptian and Saudi diabetic patients (%). * The results were significantly different between Saudi and Egyptian patients at *p*-value < 0.050.

**Figure 3:**
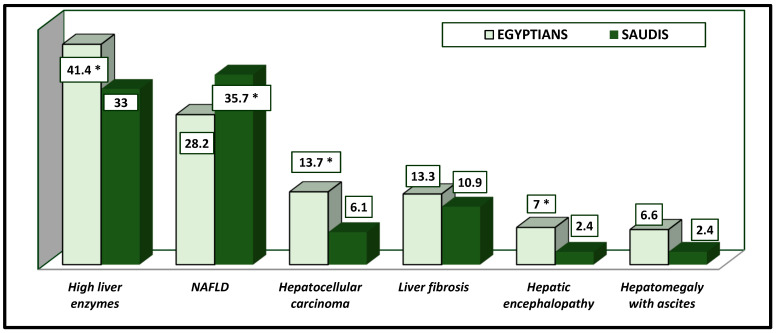
Types of liver disease due to DM Type 2 among Egyptian and Saudi patients (%). * The results were significantly different between Saudi and Egyptian patients at *p*-value < 0.050.

Regarding the impact of DM on liver diseases, about 35.5% (Saudis) vs. 23.5% (Egyptians) had liver diseases due to diabetes (Figure 2). Liver enzyme levels were increased in many Saudi and Egyptian patients (33% vs. 41.4%), as were the presence of fatty liver (35.7% vs. 28.2%) and hepatocellular carcinoma (6.1% vs. 13.7%) (Table 3 and Figure 3). All these results were significantly different between the two nations (*p*-value = 0.047).

**Figure 4:**
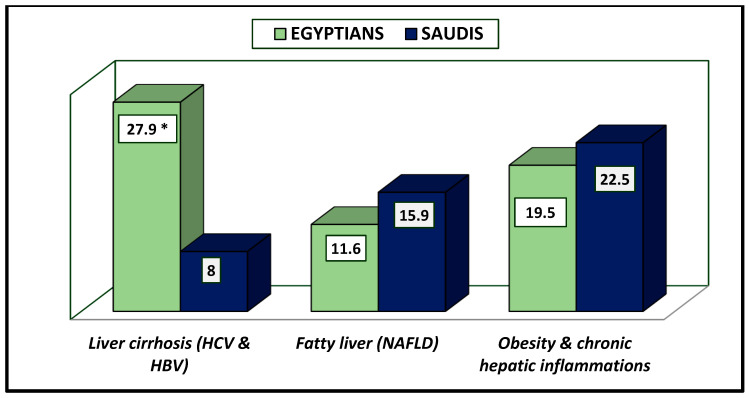
Liver diseases that lead to diabetes among Egyptian and Saudi diabetic patients (%). * The results were significantly different between Saudi and Egyptian patients with liver cirrhosis at *p*-value < 0.050.

Table 3 reveals the impact of liver diseases on DM observed in Saudi (46.4%) vs. Egyptian (59%) patients (Figure 2), showing a significant difference at *p*-value = 0.017. Liver cirrhosis due to chronic HCV or HBV was known to be a reason for diabetes in Egyptians (27.9%) vs. Saudis (8.0%), while higher incidences of fatty liver leading to DM were observed in more Saudis than Egyptians (15.9% vs. 11.6%). Obesity and chronic hepatic inflammation were highly recorded among Saudi (22.5%) vs. Egyptian patients (19.5%) (Figure 4). All results were significantly different between the two countries, at *p*-value = 0.000.

**Figure 5:**
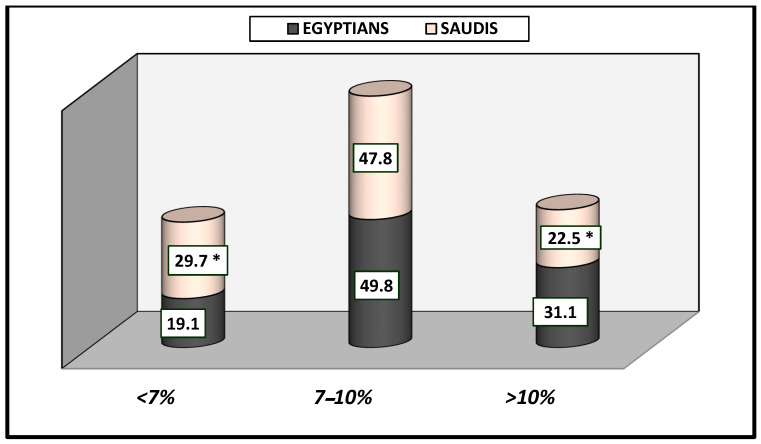
Hemoglobin HbA1c among Saudi and Egyptian patients with diabetes (%). * The results were significantly different between Saudi and Egyptian patients in HbA1c (<7% and >10%) at *p*-value < 0.050.

Table 3 shows that Saudi and Egyptian patients had elevated levels of fasting blood sugar (80.4% vs. 64.9%), which was significantly different at *p*-value = 0.001, while postprandial blood sugar levels were not significantly different at *p*-value = 0.759 (87.7% vs. 87.3%). Glycated hemoglobin level (HbA1C) was significantly different at *p*-value = 0.034; less than 7% (29.7% vs. 19.1%), from 7–10% (47.8% vs. 49.8%), and more than 10% (22.5% vs. 31.1%) were detected among Saudis and Egyptians, respectively (Figure 5).

**Figure 6:**
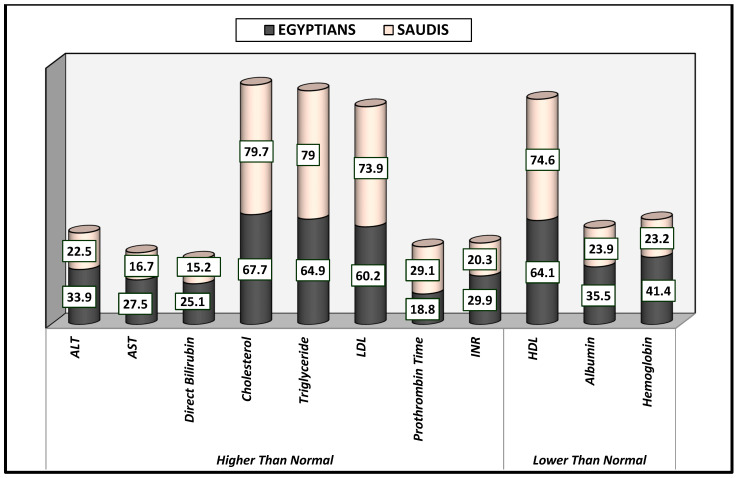
Serum level of laboratory findings among Egyptian and Saudi diabetic patients (%). All results were significantly different between Saudi and Egyptian patients at *p*-value < 0.050.

Table 4 clarifies the laboratory findings. Serum levels of alanine transaminase (ALT) and aspartate aminotransferase (AST) were found to be more elevated in Egyptian (33.9% and 27.5%) than in Saudi (22.5% and 16.7%) patients, significantly different at *p*-value < 0.050. In addition, direct bilirubin level was more elevated in Egyptian (25.1%) than in Saudi (15.2%) patients at *p*-value = 0.023 (Figure 6).

Table 4 also shows that serum levels of hemoglobin and albumin were lower in Egyptians (41.4% and 35.5%, respectively) than in Saudis (23.2% and 23.9%, respectively). More than 60% of the Egyptian patients and more than 73% of the Saudi patients showed elevated lipid profiles. The high levels of cholesterol, triglycerides, and LDL, as well as the low levels of HDL, were significantly different at *p*-value < 0.050. Also, this study revealed elevated prothrombin time and INR values to be greater in Egyptians (34.2% and 27.7%, respectively) than in Saudis (27.5% and 28.2%), significantly different at *p*-value < 0.050 (Figure 6).

## 4. Discussion

This study reveals the association between DM and LD among Saudi and Egyptian patients—both the impact of DM on the development of liver diseases and, vice versa, the impact of LD on the development of diabetes or insulin resistance.

In young Saudi patients (between 41 and 60 years old), it was observed that obesity, long duration of DM (between 10 and 20 years), unhealthy food, poor physical activity, and unadjusted lipid profiles led to a great impact of DM on the development of liver diseases in 35.5% of patients, while 46.4% of patients with liver diseases became diabetics or developed high insulin resistance.

On the other hand, among Egyptian patients (51 years old and above), it was observed that longer duration of DM (>20 years), obesity, unhealthy food, and poor physical activity (although this was better than the Saudis), and unadjusted lipid profile led to a great impact of DM on developing liver diseases in 23.5% of the patients, while 59% of patients with liver diseases became diabetics or developed high insulin resistance.

These results are similar to other studies, which found that between 2016 and 2022, 28% of the general adult population of the KSA had T2DM, and that the risks appearing in persons >40 years old were about twice as high as those aged over 40 [23]. The prevalence of T2DM was found to be high. Age of ≥40 years, male gender, and low monthly income were found to be associated with T2DM [24]. Other studies found that more prevalence was observed among older (44.6%) than younger (15.6%) people [25].

Regarding gender, more women than men were liable to have DM, especially in the KSA (58%), a significant difference, while more men were liable to have DM in Egypt (55.4%). Our results agree with three other studies in Saudi Arabia, which indicated that women had a slightly higher risk of T2DM than men [25,26,27]. Middle-aged Saudi Arabians have greater chances of developing T2DM. This could be part of the strong age-related relationship between obesity prevalence and age [28]. Obesity and overweight were prevalent among those over 40. The decline in the prevalence of obesity in adults over 70 may be due to a lower survival rate for obese individuals and a decline in physical activity with aging in both men and women. Menopausal women are likewise more prone to put on weight beyond 45 years [29]. Over the past 20 years, obesity and overweight have become more common throughout the Middle East, according to a recent study [30]. According to this report, Saudi Arabia ranked third among Middle Eastern nations for obesity, after Kuwait and Iraq. Given the high prevalence of obesity and other risk factors for non-communicable diseases, it is interesting to note that Kuwait, the United Arab Emirates, and Saudi Arabia exhibit a higher prevalence of T2DM among adults in the Gulf area, and that Saudi Arabia has the third-highest prevalence of T2DM after Kuwait and the United Arab Emirates [30,31,32].

Regarding smoking, this study showed that about one-third of diabetic patients in both nations are smokers, which agrees with other studies [33,34]. In contrast, a subsequent investigation found that weight gain after stopping smoking became a substantial risk factor for T2DM [35]. In Riyadh, KSA, 23.2% of subjects are smokers [36].

A significant percentage of Saudi patients (35.5%) endured diabetes for >10–20 years, whereas 39.8% of Egyptian patients suffered for more than 20 years. This does not agree with another study that reported that about 40% of individuals in the KSA had DM for more than ten years, compared to 12% of Egyptian participants in another study. A long duration of DM leads to depression and anxiety among both Saudi and Egyptian patients that significantly affect their work and duties [33]. About 52.4% of patients in Riyadh, KSA, were diagnosed with diabetes at the age of 40 or older, of whom 60.5% suffered from diabetes for more than 10 years, and 73% for more than five [36].

Diabetes rates worldwide will be the second highest in the Middle East and North Africa areas [37]. The prevalence of diabetes in the Arab world has increased greatly over the past two decades; this increase is attributed to various factors, including genetic predisposition, obesity, rapid urbanization, poor nutritional habits, and lack of physical activity. [38]. The findings of this study are consistent with the current literature on DM and its risk factors in both Egyptian and Saudi populations. Hegazi et al. (2016) reported a significant increase in the prevalence of T2DM in Egypt over the past two decades, attributing this rise to both traditional and unique risk factors [39]. Traditional risk factors included obesity and physical inactivity, while unique risk factors encompassed increased exposure to environmental risk factors, such as pesticides, and a high prevalence of HCV. [40] These findings align with the current study, which reported a high prevalence of T2DM and liver complications among Egyptian patients. Similarly, a rise in T2DM and associated complications has been noted in Saudi Arabia. The role of diabetes is emphasized as a risk factor for many infectious diseases, contributing to the large infectious disease burden in tropical countries, including Saudi Arabia [41]. This is consistent with the current study’s findings, which reported a high prevalence of liver complications among Saudi diabetic patients.

However, about 58% exercised for less than 60 minutes or never exercised, which may show a dislike of leading a healthy lifestyle [36]. The current study’s findings align with these observations, as a significant proportion of the studied population was found to be obese and to follow a poor healthy diet or exercise regimen. DSMES augments knowledge, glycemic control, and both clinical and psychological outcomes. It also reduces hospital admissions and all-cause mortality, while demonstrating cost-effectiveness [10].

This study has shown that nearly one-third of patients in the two nations received insulin, 21.7% received OHA only, and the other third was treated with OHA in addition to glucose regulator. We agree with a study done in 2020, which found that about 39.7% of patients took insulin either alone or in conjunction with oral hypoglycemic medicine (OHA), while nearly half of the participants used OHA alone [36].

This study also highlights the high prevalence of comorbidities among the diabetic population, particularly liver diseases. Liver diseases may occur because of diabetes, and the reverse is true. This association can be divided into the following categories: liver disease related to diabetes (diabetic hepatopathy), hepatogenous diabetes (HD), and liver diseases that occur in conjunction with diabetes [42].

Regarding the impact of DM leading to the occurrence of liver diseases, our study found that 35.5% (Saudi) and 23.5% (Egyptian) patients had liver diseases due to DM. We suggest that diabetes is prevalent among Saudis due to uncontrolled diets with more dates and carbohydrates and less exercise. Fatty liver disease and liver cirrhosis due to DM were found among Saudi and Egyptian patients. These results were in accordance with a study done in 2020, which found that about 19% of the people had diabetes, 40% of whom had fatty livers, and 39% of whom had cirrhosis, compared to 20% of people without diabetes (*p* < 0.001). Of the diabetic patients who had acute hepatitis, one-fifth developed severe or fatal hepatitis. Compared to 9% of patients with chronic hepatitis B, 16% of noncirrhotic chronic hepatitis C patients had diabetes. Diabetes affected 32% of cirrhotic patients. Cryptogenic or nonalcoholic steatohepatitis-associated cirrhosis was seen in more than 40% of patients with diabetes and cirrhosis [43]. Among our patients, some had HCC and hepatic encephalopathy. These results are in accordance with a recent study, which found a 3.9% incidence of new-onset hepatocellular carcinoma (HCC) among diabetics; over 39% of patients with HCC had diabetes, and 50% of the diabetics had HCC [43]. Nonalcoholic fatty liver disease (NAFLD) and metabolic dysfunction-associated fatty liver disease (MAFLD) were found in the population at rates of about 25% for each [44]. Diabetes is prevalent in cirrhosis of the liver (LC). There is a reciprocal pathophysiological relationship, as DM raises the rate of LC, which is itself is a diabetogenic illness [45]. Other studies have revealed that 22% of patients have diabetes before contracting liver disease [46].

In addition, regarding hepatic cirrhosis and fibrosis, Eastern Europe had the highest prevalence of metabolic dysfunction-associated steatohepatitis (MASH) among T2DM patients (about 80%), followed by the Middle East (about 70%), while Africa (about 50%) had the lowest frequency. The global pooled prevalence of MASH, substantial fibrosis, and advanced fibrosis among patients with liver biopsy data was about 66%, 40%, and 15%, respectively [47]. Another study conducted in Florida (USA) found that at least 15% of T2DM patients had moderate to advanced fibrosis, which is recognized as a risk factor for cirrhosis and overall mortality [48]. The American Diabetes Association’s recommendations to test for clinically significant fibrosis in T2DM patients with steatosis or high ALT are supported by these data [49]. About one-fifth of patients with diabetes have advanced fibrosis (a condition ten times greater than in the general population), and almost 70% have NAFLD, so it is crucial to take these patients into consideration when analyzing case results of advanced liver fibrosis and cirrhosis. Additionally, compared to those with T2DM generally, patients with alcohol use disorders had a higher rate of liver-related morbidity [50].

Another study showed that the changes observed in a diabetic patient’s liver included fatty liver, reduction in glycogen, reduced gluconeogenesis, and increased risk of liver and biliary tract cancers [51]. Our study showed a greater increase in liver enzymes among Egyptian patients than among Saudi patients because of diabetes (41.4% vs. 33%). This is confirmed by another study in Chinese patients, which found that increased levels of liver enzymes were associated with increased risks of incident diabetes [52].

Next, we look at the impact of liver diseases that develop diabetes. The long-term duration of high blood sugar may damage internal organs, including the liver. In similar ways, NAFLD and NASH raise the risk of prediabetes and T2DM. High blood sugar levels can be caused by fat accumulation and liver impairment. Some studies have showed that those with liver disease may experience a rise in blood sugar following eating a meal high in carbs [12]. During feeding, the liver absorbs glucose and uses glycolysis to oxidize it. Glycogen is first stored in the liver as surplus glucose, then lipogenesis converts it into triacylglycerols. The increased glycogen storage in the liver and the activation of glycogen synthase are both tied to insulin. Insulin plays a critical role in reducing glycogenolysis. A further crucial variable to preserve glucose homeostasis in fasting is the liver’s function through insulin counter-regulatory hormones (glucagon and epinephrine), which promote glycogen phosphorylase and glycogenolysis in the liver [53].

In the current study, it was found that around 46.4% (KSA) vs. 59% (Egypt) of patients blamed their diabetes on liver illness. In the KSA, the liver diseases that most affected DM were obesity and chronic hepatic inflammation, followed by hepatic steatosis, while in Egypt the disease was liver cirrhosis due to HCV and HBV. As with our findings, a study by Hsieh et al. (2011) showed the effects of liver disease on β cell dysfunction and insulin resistance as well as the potential mechanism by which coexisting hepatic diseases aggravate the development of T2DM [53]. Steatohepatitis and hepatic steatosis (fatty liver) are caused by fatty liver disease (FLD) [54]. Elevated liver enzymes indicate a future risk for the development of metabolic syndrome and insulin resistance [55]. Diabetes risk has been demonstrated to be elevated in cases of alcoholic liver disease. Heavy alcohol consumers with FLD or moderate alcohol consumers without FLD had a significantly increased risk for emerging T2DM, compared with no alcohol consumers without FLD [56]. A study showing that liver fat concentration influences insulin sensitivity in people far more than visceral fat mass does suggests that fatty liver plays a direct and significant role in the pathophysiology of insulin resistance [57,58]. A specific investigation has been done on the impact of fatty liver’s inflammatory changes on the development of β cell dysfunction in T2DM. It showed that a reduction in pancreatic insulin secretion existed with an inflammatory liver produced by mild portal endotoxemia. The most frequent correlations between diabetes and liver diseases are diabetic hepatosclerosis, HD, glycogenic hepatopathy, and NAFLD [59]. The close connection between cirrhosis and poor glycemic control is referred to as HD. A recent study showed that about 36% of liver cirrhosis patients were found to have impaired glucose tolerance and 25% to have T2DM. This, in turn, may play a role in the development of insulin resistance through the downregulation of insulin receptors. However, rather than the development of cirrhosis alone, the interaction of the cause of liver cirrhosis with environmental factors may also play a key role in the relationship between cirrhosis and diabetes [60]. Other research has proven the impact of liver diseases on developing diabetes. It was revealed that T2DM is more common in patients with chronic HCV and HBV infections than in other populations [61]. It has been suggested that HCV may affect glucose homeostasis, and abnormal glycol-metabolism was found to be prevalent in patients with HCV infection. After viral elimination, glycol-metabolism improved [62].

## 5. Conclusions

This study provides valuable insights into the characteristics and challenges faced by diabetic patients with liver diseases in Egypt and Saudi Arabia. We found that the majority of diabetic patients suffer bad effects on the development of liver disorders (more among Saudi patients, mainly due to a higher prevalence of obesity), and that most liver diseases exert bad impacts on the progress of DM (especially in Egyptians, mainly due to liver cirrhosis caused by HCV and HBV). There is a link between diabetes and liver diseases. In particular, liver cirrhosis and diabetes were found to influence each other. Therefore, the right medication, lifestyle modifications, successful cirrhosis control (in patients with liver diseases), and successful diabetic control (in diabetic patients) could lead to the effective management of both diseases. The negative fallouts in the two cases were prompted by obesity, morbid eating, and poor quality of life. Healthcare providers should think about using specific strategies and targeted interventions to care for patients with both diabetes and liver diseases to obtain better patient outcomes and quality of life. We recommend further research to explore the underlying reasons for the observed trends and to develop effective strategies to address them.

## Figures and Tables

**Table 1:** Flow chart of study participants.

Total number of patients with diabetes: 2873 (1295 Saudi and 1578 Egyptian).		
Saudi Patients (1295)		Egyptian Patients (1578)
Exclusion for age less than 20 years-old	18	22
Exclusion for incomplete data	35	50
Total Exclusion	53	72
Number of patients that met inclusion criteria	1242	1506

**Table 2:** The socio-demographic characteristics of Egyptian and Saudi diabetic patients (%) [Total n = 2748 n = 1242 (Saudis), n = 1506 (Egyptians)].

Characteristics (%)	Saudis [1242]	Egyptians [1506]	Total [2748]	*p*-Value
Gender	n (%)	n (%)	n	
Male	522 (42.0)	834 (55.4)	1356	0.010 *
Female	720 (58.0)	672 (44.6)	1392	
**Age (years)**				
**Age (Mean ± SD)**	50.96 ± 17.53	57.13 ± 7.25		≤0.050 *
**Age**	**n (%)**	**n (%)**	**n**	
20–30	135 (10.9)	144 (9.6)	279	0.000 *
31–40	153 (12.3)	150 (10.0)	303	
41–50	441 (35.5)	240 (16.0)	681	
51–60	198 (15.9)	420 (28.0)	618	
61–70	180 (14.5)	324 (21.6)	504	
>70	135 (10.9)	228 (15.1)	363	
**Duration of diabetes**				
<5 years	243 (19.6)	150 (10.0)	393	0.000 *
5–10 years	297 (23.9)	348 (23.1)	645	
>10–20 years	441 (35.5)	408 (27.1)	849	
>20 years	261 (21.0)	600 (39.8)	861	
**Smoking**				
Yes	414 (33.3)	468 (31.1)	882	0.016 *
No	828 (66.7)	1038 (68.9)	1866	
**Obesity**				
Yes	792 (63.8)	732 (48.6)	1524	0.019 *
No	450 (36.2)	774 (51.4)	1224	
**Following healthy diet**				
Yes	378 (30.4)	774 (51.4)	1152	0.022 *
No	864 (69.6)	732 (48.6)	1596	
**Physical activities and fitness**				
Yes	459 (37.0)	768 (51.0)	1227	0.036 *
No	783 (63.0)	738 (49.0)	1521	
**Effect of** diabetes **on work and activity**				
Yes	909 (73.2)	996 (66.1)	1905	0.029 *
No	333 (26.8)	510 (33.9)	843	

* Significant difference at *p* ≤ 0.05.

**Table 3:** The clinical characteristics of Egyptian and Saudi diabetic patients (%) [Total n = 2748 n = 1242 (Saudis), n = 1506 (Egyptians)].

Characteristics Number = n	Saudis [1242]	Egyptians [1506]	Total [2748]	*p*-Value
Treatment of DM	n (%)	n (%)	n	
Insulin Injection	414 (33.3)	492 (32.7)	906	0.028 *
OHA only	270 (21.7)	474 (31.5)	744	
DR only	162 (13.1)	84 (5.5)	246	
OHA + DR	396 (31.9)	456 (30.3)	852	
**Impact of DM on liver diseases**				
Yes	441 (35.5)	354 (23.5)	795	0.011 *
No effect on liver diseases	801 (64.5)	1152 (76.5)	1953	
**Type of liver** **diseases due to DM**				
High liver enzymes	410 (33.0)	624 (41.4)	1034	0.047 *
Liver fibrosis	135 (10.9)	200 (13.3)	335	
Hepatomegaly with Ascites	30 (2.4)	100 (6.6)	130	
Hepatocellular carcinoma (HCC)	76 (6.1)	207 (13.7)	283	
Fatty liver (NAFLD)	443 (35.7)	425 (28.2)	868	
Hepatic encephalopathy	30 (2.4)	105 (7.0)	135	
No effect on liver diseases	801 (64.5)	1152 (76.5)	1953	
**Impact of liver diseases on DM**				
Yes	576 (46.4)	888 (59.0)	1464	0.017 *
No effect on DM	666 (53.6)	618 (41.0)	1284	
**Type of liver disease developing DM**				
Liver cirrhosis (HCV, HBV)	99 (8.0)	420 (27.9)	519	0.000 *
Fatty liver (NAFLD)	198 (15.9)	174 (11.6)	372	
Obesity & chronic hepatic inflammation	279 (22.5)	294 (19.5)	573	
No effect on DM	666 (53.6)	618 (41.0)	1284	
**Fasting Blood Sugar**				
Normal	243 (19.6)	528 (35.1)	625	0.001 *
High	999 (80.4)	978 (64.9)	2123	
**Postprandial Blood Sugar (after 2 h)**				
Normal	153 (12.3)	192 (12.7)	345	0.759
High	1089 (87.7)	1314 (87.3)	2403	
**Hemoglobin (HbA1C)**				
<7%	369 (29.7)	288 (19.1)	657	0.034 *
7–10%	594 (47.8)	750 (49.8)	1344	
>10%	279 (22.5)	468 (31.1)	747	

* Significant difference at *p* ≤ 0.05. OHA: Oral hypoglycemic agents; DR: Diabetes regulators.

**Table 4:** Laboratory findings among Egyptian and Saudi patients (%) [Total n = 2748 n = 1242 (Saudis), n = 1506 (Egyptians)].

Characteristics (%)	Saudis [1242]	Egyptians [1506]	Total [2748]	*p*-Value
**Alanine transaminase (ALT)**	**%**	**%**	**n**	
Normal	963 (77.5)	996 (66.1)	1959	0.019 *
High	279 (22.5)	510 (33.9)	789	
**Aspartate aminotransferase (AST)**				
Normal	1035 (83.3)	1092 (72.5)	2127	0.016 *
High	207 (16.7)	414 (27.5)	621	
**Direct Bilirubin**				
Normal	1053 (84.8)	1128 (74.9)	2181	0.023 *
High	189 (15.2)	378 (25.1)	567	
**Albumin**				
Normal	945 (76.1)	972 (64.5)	1917	0.019 *
Low	297 (23.9)	534 (35.5)	831	
**Hemoglobin**				
Normal	954 (76.8)	882 (58.6)	1836	0.000 *
Low	288 (23.2)	624 (41.4)	912	
**Cholesterol level**				
Normal	252 (20.3)	486 (32.3)	738	0.012 *
High	990 (79.7)	1020 (67.7)	2010	
**Triglyceride level**				
Normal	261 (21.0)	528 (35.1)	789	0.004 *
High	981 (79.0)	978 (64.9)	1959	
**LDL (low density lipoprotein)**				
Normal	324 (26.1)	600 (39.8)	924	0.006 *
High	918 (73.9)	906 (60.2)	1824	
**HDL (high density lipoprotein)**				
Normal	315 (25.4)	540 (35.9)	855	0.034 *
Low	927 (74.6)	966 (64.1)	1893	
**PT (prothrombin time)**				
Normal	1008 (81.2)	1068 (70.9)	2076	0.026 *
High	234 (18.8)	438 (29.1)	672	
**INR**				
Normal	990 (79.7)	1056 (70.1)	2046	0.040 *
High	252 (20.3)	450 (29.9)	720	

* Significant difference at *p* ≤ 0.05.

## Data Availability

All data are available within the article.
